# First order derivative spectra to determine caffeine and chlorogenic acids in defective and nondefective coffee beans

**DOI:** 10.1002/fsn3.1723

**Published:** 2020-07-27

**Authors:** Daniel Habtamu, Abebe Belay

**Affiliations:** ^1^ Department of Applied Physics Adama Science and Technology University School of Applied Natural Sciences Adama Ethiopia

**Keywords:** caffeine, chlorogenic acid, defective and nondefective, first derivative

## Abstract

In this research, the application of the first order derivative spectra was employed to determine the levels of caffeine (CAF) and chlorogenic acids (CGA) in defective (immature, black, and sour) and nondefective coffee beans without using extraction or background correction techniques. The extreme points of first order derivate spectra of these compounds were at the wavelength of 260 and 292 nm enable to quantify the contents of CAF and CGA, respectively. The level of CAF and CGA in coffee beans determined by this method is ranged from 1.2 ± 0.12–1.46 ± 0.47% and 4.04 ± 0.44–4.43 ± 0.43%, respectively. The study results also indicated total contents of CAF and CGA levels discriminate the defective and nondefective coffee beans with higher CAF and CGA contents being observed in defective coffee beans. As the method is extremely rapid, easy, and inexpensive and also requires minimal sample preparation for the quantification of CAF and CGA contents in coffee, it could be a valuable quality control technique.

## Graphical abstract

First order derivative spectra to determine CGA and CAF in coffee beans.

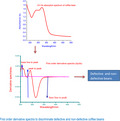

## 1 INTRODUCTION

On a global scale, coffee is one of the most heavily consumed beverages, with an average daily consumption of 2.3 billion cups per day (Higdon & Feri, 2006). However, there is lack of quality coffee supply to market due to pre‐ and postharvesting processes. These defective beans are damped for internal market instead of exported to different countries (Craig, Franca, & Oliveira, 2012). Currently, it represents 20% of the total coffee produced in Brazil (Mazzafera, 1999; Ramalakshmi, Kubra, & Rao, 2007). In Ethiopia, the percentage of defected beans are higher than other countries (Belay et al., 2014).

Each coffee producing country has own means of discriminate defect beans. Usually coffee quality assessments were performed by cupping techniques. However, such evaluation techniques are highly subjective and not reliable. In addition, cupping is time‐consuming and dependent on trained cupper experts (Feria, 2002). Thus, there is a need to develop fast and reliable scientific methods to assess the quality of coffees before or after roasting.

In the past years, several studies have been conducted on the defective coffee beans (Franca, Oliveira, Mendonca, & Silva, 2005). These studies are shown that there is difference in the physical and chemical properties between immature, black, sour, and nondefective coffee beans. Recent study showed the physical property alone can successfully separate defective and nondefective beans (Belay et al., 2014). Other studies (Mendonça, Franca, & Oliveira, 2009) also applied sieving for separation of these beans. Some other studies (Craig, Franca, & Oliveira, 2011; Craig et al., 2012 Farah, Monteiro, Calado, Franca, & Trugo, 2006; Franca et al., 2005; Ramalakshmi et al., 2007), on the other hand attempted to employ direct diffused reflectance Fourier‐transform infrared spectroscopy and high‐performance liquid chromatography (HPLC) methods to determine level of caffeine (CAF) and chlorogenic acids (CGA) to discriminate the defective and nondefective beans.

The chemical techniques are powerful in quantifying these bioactive compounds; however, it needs expensive instruments and solvents. In addition, CAF or CGA should be extracted from coffee solution before measurement. These can be performed by extraction and isolation of CAF and CGA using organic solvents like benzene, chloroform, and dichloromethane (Zhang, Lian, Wang, & Chen, 2005), which are harmful for health and environment. However, in this research, first order derivative spectra applied for the determination of CAF and CGA in defective and nondefective coffee beans without applying extraction and preseparation techniques.

Previously study by Alpdogan, Kariban, and Sungur (2002), Sanchez, Bosh, Jeda, and Cano (1988), Savitzky and Golay (1964), and Talsky (1994) try to determine the contents of CAF in nondefective coffee beans using derivative spectrophotometry, on the other hand, to the author's knowledge, simultaneous determination of the level of CAF and CGA using first order derivative spectra for the assessment of coffee quality has not been previously described in any literature. Hence, as inexpensive and simple method apply for the analysis of these bioactive compounds in defective and nondefective coffee beans are useful. Therefore, the objective of the present study was to apply first order derivative spectra method for analysis of CAF and CGA for the discrimination purpose.

## 2 MATERIALS AND METHODS

### 2.1 Chemicals and samples

**FIGURE 1:**
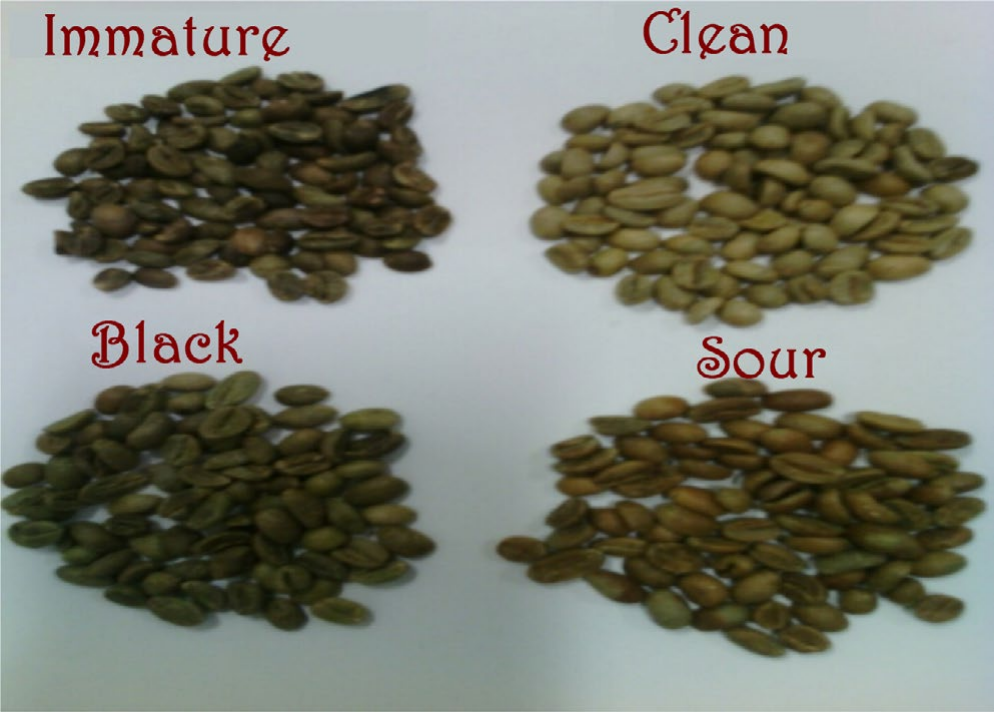
Coffee beans sorted into four lots: defective (immature, black, and sour) and nondefective

Caffeine (Evan) and CGA (Aldrich‐Sigma) were used for preparation of standard solution. To determine the level of CAF and CGA in defective and nondefective, the samples were collected from three areas of Oromia regional state (Ethiopia): namely Geri, Sorgeba, and Nano Chala. From each area, a mass of 300 g of coffee samples (batch 2012/2013) was randomly collected and subjected to sorting into defective (*full black*, *full sour*, and *immature*) and nondefective beans by senior coffee quality expert. Figure 1 shows green coffee beans sorted into four lots: defective *(immature*,* black*, and* sour*) and nondefective.

### 2.2 Apparatus and instrumentation

The following laboratory apparatus were used for the experiment—magnetic stirrer, separatory funnel, quartz cuvette, and 250 µm sieve. For ultraviolet–visible (UV‐Vis) absorption spectral measurement of CAF, CGA and samples, UV‐Vis‐near infrared spectroscopy (Perkin Elmer) were used. It was operated by UVCSS software. Spectral derivative has been performed according to the method developed by (Alpdogan et al., 2002; Sanchez et al., 1988; Savitzky & Golay, 1964; Talsky, 1994).

### 2.3 Methods of the experiment

#### 2.3.1 Calibration curve for the standard solution of CAF and CGA

Caffeine of 0.05 g/ml stock solution was prepared and diluted to obtain different concentrations (4, 5, 6, and 7 µg/ml). Absorption spectra of the standard solutions were recorded in the wavelength of 200–400 nm. Similarly, CGA of 0.06 g/ml were diluted (7, 8, 9, and 10 µg/ml) to prepare calibration curves. The UV‐Vis absorption spectra of CGA standard were in the wavelength of 200–500 nm.

The first order derivative spectra were obtained and its absorption spectra versus concentration were plotted using “baseline‐to‐peak” measurement technique according to calibration curve already developed by other workers (Alpdogan et al., 2002). By similar procedures, CGA spectra were also recorded. Linear curve fit was applied to find linear regression equations for the standard solutions (CAF and CGA).

#### 2.3.2 Preparation of coffee samples and determination of contents of CAF and CGA from the calibration curves

0.02 g of sieved ground raw coffee was added to 50 ml of distilled water in a beaker, and the mixture was stirred for 1 hr using magnetic stirrer at room temperature. The UV‐Vis absorption spectra of each sample measured using UV‐Vis spectroscopy and their corresponding first order derivative spectra obtained. The extreme point of the wavelength of 256 and 292 nm used as the absorption peaks for CAF and CGA, respectively. The concentrations of CAF and CGA in coffee beans were deduced from the linear regression equation of the related curve using extreme points of the first order derivative spectra.

## 3 RESULTS AND DISCUSSION

### 3.1 UV‐Vis spectra of CAF and its first order derivative spectra

**FIGURE 2:**
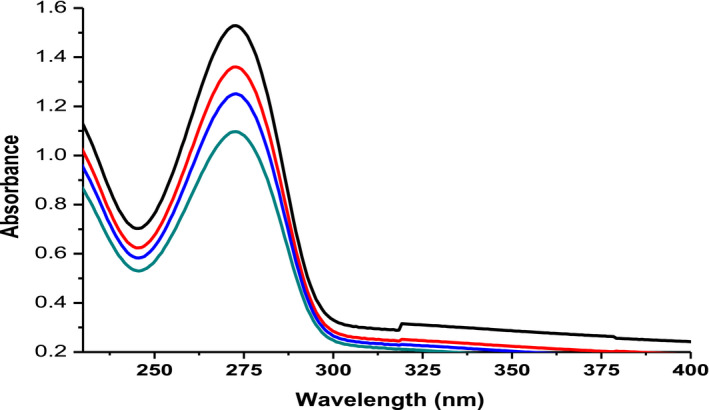
UV‐Vis absorption spectra of caffeine at different concentrations (4–7 µg/ml) in water

Figure 2 shows the absorbance spectra versus wavelength CAF measured at different concentrations (4, 5, 6, and 7 µg/ml). The UV‐Vis absorption spectra of CAF in water are found to be in the region of 200–400 nm. Its peak absorbance in water solution was observed at the *λ*_max_ = 272.8nm. The maximum peak absorbance for CAF observed in this experiment was quite similar with those reported previously (Belay, Ture, Redi, & Asfaw, 2008).

**FIGURE 3:**
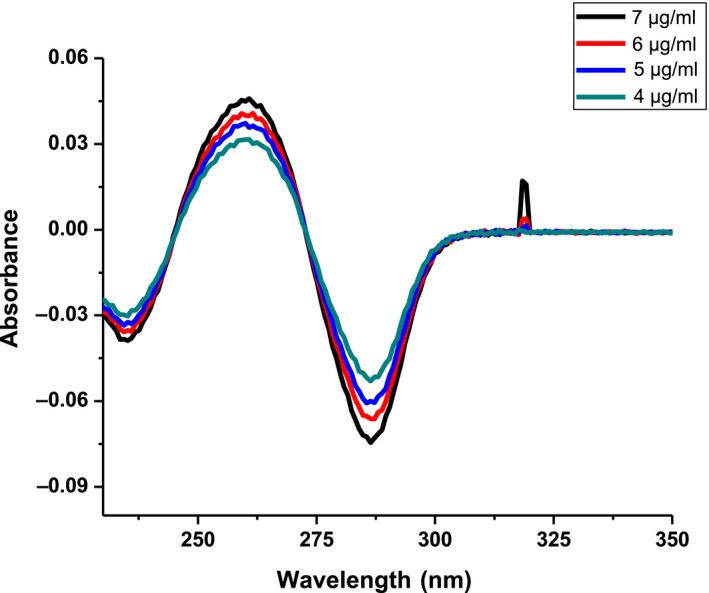
First order derivative spectra of caffeine standards at different concentrations

The first order derivative spectra of CAF were shown in Figure 3. The peak absorbance was found to be at *λ*_max_ = 260 nm shifting to the left by 12 nm. Baseline‐to‐peak measurement at the peak wavelength *λ*_max_ = 260 nm was used for preparation of calibration curve. Linear relationships were obtained between peak amplitude (*y*) of first order spectra at the extreme wavelengths and CAF concentration. The regression equation was (*y* = 0.000439*x* + 0.000975, *R* ^2^ = 0.99), *y*‐represent the peak position at *λ* = 260 nm and *x*‐concentration in µgm/ml. Linear regression equation of the first order derivative spectra was used to calculate the concentration of CAF in coffee beans.

### 3.2 Analysis of CAF in defective and nondefective by first order derivative spectra

Direct measurement of CAF in coffee beans cannot possible in the wavelength regions 200–400 nm due to spectral interference (Belay et al., 2008). The concentrations of CAF calculated from the regression equation and the results were shown in Table 1.

**TABLE 1:** Caffeine (CAF) content in defective and nondefective coffee samples obtained using first order derivative spectra and literature values

Coffee samples	CAF contents (%)	
	The present study values	Literature values
Immature	1.46 ± 0.47	0.96–1.23 (Farah et al., 2006)
Black	1.34 + 0.34	1.19–1.44 (Purcarea et al., 2008)
Sour	1.34 + 0.34	1.19–1.44 (Purcarea et al., 2008)
Nondefective	1.20 + 0.20	0.6–1.9 (Clarke & Macarae, 1985)

As shown in Table 1, the mean percentage of CAF in coffee beans are 1.46 ± 0.47 for immature, 1.34 ± 0.34 for black and sour, and 1.20 ± 0.20 for nondefective, respectively. The highest amount of CAF contents was found in immature coffee sample while the lowest level was found in the nondefective coffee sample as shown in the table. Generally, the level of CAF in the four coffee samples (*immature*, *black*, *sour*, and nondefective) has been found in the ranges of 0.6%–1.90% which is similar with previous researchers. The reported values based on the spectrophotometric determination of CAF in coffee beans by extracting using dichloromethane and chloroform are 1.20%–1.48%, 1.19%–1.44%, respectively (Purcarea, Chis, Vicas, & Fodor, 2008).

Furthermore, the determined CAF level by the present study is in agreement with the CAF contents reported using various analytical techniques. The highest and lowest CAF content for green Arabic coffee using HPLC has been reported as 1.23 ± 0.06 and 0.96 ± 0.01% respectively (Farah et al., 2006). By derivative spectrophotometry techniques, the content of CAF determined in coffee was found to be 1.36 ± 0.03% (Alpdogan et al., 2002).

The results on Table 1 also showed that there was no change in CAF contents between black and sour coffee samples (1.34 ± 0.34%), which is in agreement with the previously reported results which found no significant change on CAF contents between defective coffee beans (Clarke & Macarae, 1985; Mazzafera, 1999). In addition, other studies revealed that nondefective beans exhibited lower CAF levels than defective ones. Moreover, it was also proposed that the percentage of CAF for Arabic coffees was on the average <1.5%. From the stated literature reports, it can be inferred that the results in the present study are in the documented range of the previous study results.

The contents of CAF and coffee cup quality are still somehow controversial. Previous research reports using a UV‐Vis spectrophotometer methodology indicated that the concentrations of CAF were higher in high quality (nondefective) coffees in comparison with low quality (defective) ones. Nonetheless, the concentrations of CAF in the present study were found to be higher in low quality coffees (*immature*, *black* and *sour*) in comparison with good quality one (nondefective) in agreement previous report.

### 3.3 UV‐Vis absorption spectra CGA and its first order derivative spectra

**FIGURE 4:**
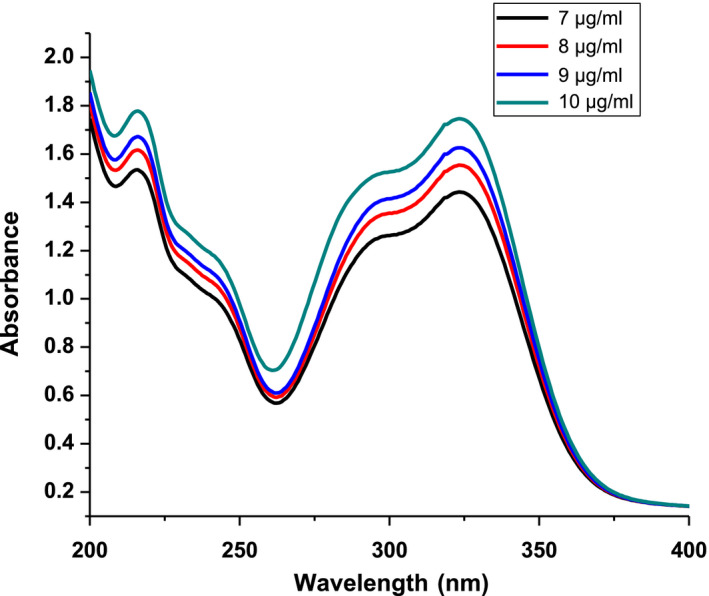
UV‐VIS absorption spectra of chlorogenic acids at different concentrations

Figure 4 shows the UV‐Vis absorption spectra of CGA in the 200–500 nm. In these regions, CGA has two peaks; the firs being at 216.8 nm and the second at 324 nm peak belongs to the HOMO → LUMO transition mainly due to ᴨ → ᴨ* transition (Cornard, Lapouge, Dangleterre, & Allet‐Bodelot, 2008) and the minimum point was at 262.5 nm as reported previously by (Belay & Gholap, 2009).

**FIGURE 5:**
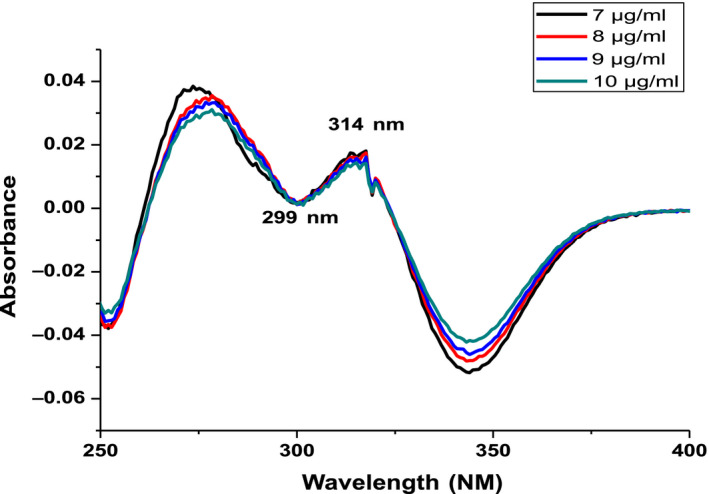
First order derivative spectra of chlorogenic acids standards in water at different concentrations

First order derivative spectra of CGA were shown in Figure 5. Baseline‐to‐peak measurement of peak wavelength *λ*_max_ = 292 nm was used for preparation of calibration curve. Linear relationships were obtained between the CAF concentration and peak amplitude (*y*) of first order spectra. The regression equation (*y* = 0.0.14036*x* + 0.053628, *R* ^2^ = 0.98) was obtained, where *y*‐represent the peak height and *x*‐concentration in µgm/ml.

### 3.4 Determination of level of CGA in defective and nondefective beans using first order derivative spectra

Directly determination of CGA in coffee is impossible due to UV‐absorbing matrix (Belay & Gholap, 2009; Zhang et al., 2005). In order to eliminate this difficulty, background correction was used. In contrast, the first order derivative allows the elimination of matrix effects because the variable background absorptions overlapping the analyte peaks are smoother in derivative spectra. Figure 5 the first order derivative spectra of CGA in water at different concentrations. The concentrations of CGA calculated using the regression equation shown in Table 2.

**TABLE 2:** The content chlorogenic acid (CGA) in defective and nondefective coffee samples determined using first order derivate spectra and literature values

Coffee samples	CGA contents (%)	
	The present study values	Literature values
Immature	4.43 ± 0.43	1.3–8.7 (Farah et al., 2005)
Black	4.26 ± 0.38	5.5–8.0 (Clarke & Macarae, 1985)
Sour	4.26 ± 0.38	6.05–6.25 (Belay & Gholap, 2009)
Nondefective	4.07 ± 0.07	4.1–7.9 (Farah et al., 2006)

The mean percentage of CGA in coffee beans investigated are 4.43 ± 0.43 for immature, 4.26 ± 0.38 for black, 4.26 ± 0.38 for sour, 4.07 ± 0.07 for nondefective, respectively. The highest amount of CGA was found in immature coffee sample while the lowest was found in the nondefective coffee sample as reported by (Clifford & Wilson, 1987). Generally, the level of CGA determined by this method is similar with the result reported by (Farah, Paulis, Trugo, & Martin, 2005; Ky, Noirt, & Hamon, 1997; Ky et al., 2001) for total CGA in regular green coffee beans which vary from 4.0% to 8.4% for coffee Arabica.

The study result also indicated the total CGA levels have an inverse association with coffee quality with higher CGA content being observed in lower quality samples (defective beans) in agreement with reports by (Farah et al., 2005) who observed a strong inverse association between the level of CGA and low cup quality. Moreover, after analysis of eight CGA isomers in defective coffee beans, the authors also observed that immature and black defective beans contained significantly higher levels of all CGA isomers, particularly CQA and FQA, compared with the nondefective beans in a good agreement with the present study. Similarly, the study result of (Mazzafera, 1999) of the comparison of immature and black beans with nondefective observed that the total CGA contents were higher in immature and immature‐black defective. Thus, the previous reports and the present results revealed that the CGA content has an inverse relationship with coffee quality samples considering that CQA accounts for at least 60% of CGA contents in coffee, higher levels of would be more likely to be associated with low cup quality.

## 4 CONCLUSIONS

First order derivative spectra employed as a methodology for the determination of CAF and CGA in defective and nondefective coffee beans. The results confirm that the derivative spectra method can be very good techniques for the elimination of interfering matrices and enable us to determine the contents of CAF and CGA in defective and nondefective coffee beans without any background correction or reagent. Considering the results obtained and presented, it can be concluded that the developed method was simple, fast, and low‐cost methods can be used to provide basic analysis of CAF and CGA contents of coffee beans. It can provide basic characterizations defective and nondefective beans before shipments to markets. A valuable noted from these results were the fact that CAF and CGA were the major compounds strictly related to coffee quality, with high levels observed for defective (*immature*,* dark*, and* sour*) and low levels for nondefective or clean coffee beans.

## CONFLICT OF INTEREST

The authors declare that they do not have any conflict of interest.

## ETHICAL STATEMENTS

Ethical review: This study does not involve any human or animal testing. Informed consent: Written informed consent was obtained from all study participants.
